# Development of an Innovative, Carrier-Based Dry Powder Inhalation Formulation Containing Spray-Dried Meloxicam Potassium to Improve the In Vitro and In Silico Aerodynamic Properties

**DOI:** 10.3390/pharmaceutics12060535

**Published:** 2020-06-10

**Authors:** Edit Benke, Árpád Farkas, Piroska Szabó-Révész, Rita Ambrus

**Affiliations:** 1 Institute of Pharmaceutical Technology and Regulatory Affairs, Interdisciplinary Excellence Centre, University of Szeged, 6720 Szeged, Hungary; benke.edit@pharm.u-szeged.hu (E.B.); revesz@pharm.u-szeged.hu (P.S.-R.); 2 Centre for Energy Research, Hungarian Academy of Sciences, 1121 Budapest, Hungary; farkas.arpad@energia.mta.hu

**Keywords:** inhalation, pulmonary drug delivery, dry powder inhaler, carrier-based DPI, meloxicam potassium, magnesium stearate, interparticle interactions, in vitro lung model, aerodynamic properties, in silico assessment

## Abstract

Most of the marketed dry powder inhalation (DPI) products are traditional, carrier-based formulations with low drug concentrations deposited in the lung. However, due to their advantageous properties, their development has become justified. In our present work, we developed an innovative, carrier-based DPI system, which is an interactive physical blend of a surface-modified carrier and a spray-dried drug with suitable shape and size for pulmonary application. Meloxicam potassium, a nonsteroidal anti-inflammatory drug (NSAID), was used as an active ingredient due to its local anti-inflammatory effect and ability to decrease the progression of cystic fibrosis (CF) and chronic obstructive pulmonary disease (COPD). The results of the in vitro and in silico investigations showed high lung deposition in the case of this new formulation, confirming that the interparticle interactions were changed favorably.

## 1. Introduction

The delivery of active pharmaceutical ingredients (APIs) via the lung allows the treatment of several local (e.g., cystic fibrosis—CF—and chronic obstructive pulmonary disease—COPD) and systemic diseases (e.g., diabetes mellitus and agitation associated with schizophrenia). This possibility can be explained by the fact that the lung has highly absorptive tissue with a large surface area, which has a thin adsorption membrane and excellent blood supply. Through this administration route, a lower dose is required compared to oral administration; thus, a more favorable side-effect profile can be achieved, and even the onset of the action could be faster [1,2,3,4].

The traditional, carrier-based dry powder inhalers (DPIs) are the most widely used formulations in this therapy, which means an interactive physical mixture of large carrier particles and particle size-optimized drugs [5,6]. The formulations produced by milling and sieving, etc., could be advantageous for those drugs that have high cohesion properties or low doses [7]. However, it should also be noted that the carrier-based DPI formulations still have only 20–30% of the fine particle fraction (FPF) values [8].

In the case of carrier-free systems, various technological processes (e.g., spray drying, spray freeze drying, and supercritical-fluid technology) and different excipients could be applied, which resulted in beneficial aerodynamic properties [9,10]. However, their disadvantages could be the swelling of the API particles during storage, as well as aggregation because of the high cohesive forces between the small particles [7,11].

Many successful results have already been published in connection with the development of traditional, carrier-based formulations. One of these is the recrystallization of the large carrier particles from the solvent, where several technological methods have been described [12,13,14,15]. Partially dissolving the surface of the carrier is another possible way to change the surface properties of the carriers [16,17]. The third possible method is to use the Wurster coating technique—a fluidized bed—to coat the carrier, e.g., with a solution of hydroxypropyl methylcellulose [18]. In addition to the wet surface modification methods, a dry coating could be applied mechanically on the surface of the carrier particles. Due to the mechanical forces during the coating, the van der Waals interactions are strong enough to bind the small particles onto the surface of the large carriers. The process is affected by the coating time, the content of the coating particles, and the properties of the large carrier [19]. The mechanical dry coating should be prepared via using a Turbula mixer or by using MechanoFusion^®^, Hybridizer^®^, Magnetically Assisted Impaction Coater, and Theta-composer^®^. During this mechanical dry coating method, fine carriers, sucrose tristearate, and magnesium stearate (MgSt) are used frequently [20,21,22]. The previously mentioned methods containing MgSt are already applied by many marketed products, e.g., by Foster^®^ NEXThaler^®^ (Chiesi Farmaceutici, Parma, Italy) and Relvar^®^ Ellipta^®^ (GSK Pharmaceuticals Ltd., Brentford, UK) inhalation powders [23].

The abovementioned procedures of DPI development could be combined. For example, initially, Adi et al. [24] used a mixture of an untreated carrier and spray-dried drug, Hazare et al. [25] carried out manual mixing as a surface treatment between the lactose carrier and the MgSt, and then mixed them with a spray-dried API. Fualhammer et al. [26] blended the decanted engineered lactose and the spray-dried drug, and Mönckedieck et al. [27] applied spray-dried API particles and mannitol on the surface of larger spray-dried mannitol (~60 µm), etc. However, the FPF values were lower than 50%. It should be noted that comparisons of FPF values should be done carefully because different types of in vitro testing equipment, airflows, DPI devices, and capsules could cause some differences in the FPF results. In our previous work we also successfully produced innovative, carrier-based, antibiotic-containing DPI formulations. Through these formulations, the spray-dried/co-spray-dried drug particles were applied onto the surface of an MgSt-coated carrier (the size of the raw MgSt was D (0.5): 6.92 µm) to achieve an outstanding ~70% of the FPF values [28,29].

The effect of nonsteroidal anti-inflammatory drugs (NSAIDs) in the lung is a direct reduction in inflammation and an indirect decrease in the progression of the disease. In CF and COPD, a similar pathophysiological cycle occurs during the progression of the disease [30], which slowly leads to respiratory failure [31,32,33,34,35,36]. In COPD, it has been confirmed that cigarette smoke, in addition to causing inflammation, degrades CFTR expression, thereby triggering an ‘‘acquired’’ CFTR dysfunction similar to the pathophysiological cycle described for CF [32]. Furthermore, NSAIDs could be used in a non-small cell lung cancer (NSCLS) as adjunctive therapies, because COX-2 is also overexpressed in NSCLS, thereby applying the COX-2 inhibitors can slow down the progression of malignant tumors [37].

Nevertheless, there is no marketed product with NSAIDs for pulmonary administration. However, more and more articles could be found dealing with pulmonary NSAID delivery [37,38,39,40,41,42,43,44,45,46]. The physicochemical properties of the APIs play an essential role in the formulation of pulmonary powders, so it is advisable to work with a water-soluble salt form. For example, meloxicam potassium (MXP), patented by Egis Pharmaceuticals Ltd., is a pure intermediate of meloxicam (MX), whose water solubility (13.1 mg/mL at 25 °C) far exceeds MX (4.4 μg/mL at 25 °C) and has hundreds of times better solubility at bronchial pH (pH 7.4) than compared with MX [47,48,49]. Using MX, compact microcomposites could be produced [50], and the application of large and porous MXP particles with improved properties was subsequently developed [43] by our research group.

The possibility to modify the surface of the carrier by a high-shear mixing technique is strongly dependent on the adjuvant used in the smoothing process. The smoothing adjuvant (e.g., MgSt, isoleucine, and arginine) used to coat the carrier has a certain influence on its respirability [51]. Therefore, this work aims to develop an innovative, carrier-based DPI containing MXP, using the advantages of carrier-free and carrier-based techniques, and where we applied a spray-dried API blended with a carrier or surface-modified carrier to achieve improved lung deposition. On the other hand, our work confirms that our previous development, using an antibiotic agent, can be successfully performed for MXP (as an NSAID drug) as well, and meet the requirements of a DPI. So, we proved the achievement of an innovative formulation that can contribute to the improvement of CF and COPD therapy in the future.

## 2. Materials and Methods

### 2.1. Materials

MXP, a novel water-soluble salt (Egis Pharmaceuticals PLC, Budapest, Hungary), was applied as an active ingredient. Lactose monohydrate Inhalac^®^ 70 (IH70), as a large carrier, was kindly supplied by the MEGGLE Group (Wasserburg, Germany). MgSt (D (0.5): 6.92 µm) (Sigma Aldrich, Budapest, Hungary) was chosen as the surface-modifying agent, and 96% ethanol was obtained from AppliChem GmbH (Darmstadt, Germany).

### 2.2. Methods

#### 2.2.1. Preparation of the Formulations

**Figure 1:**
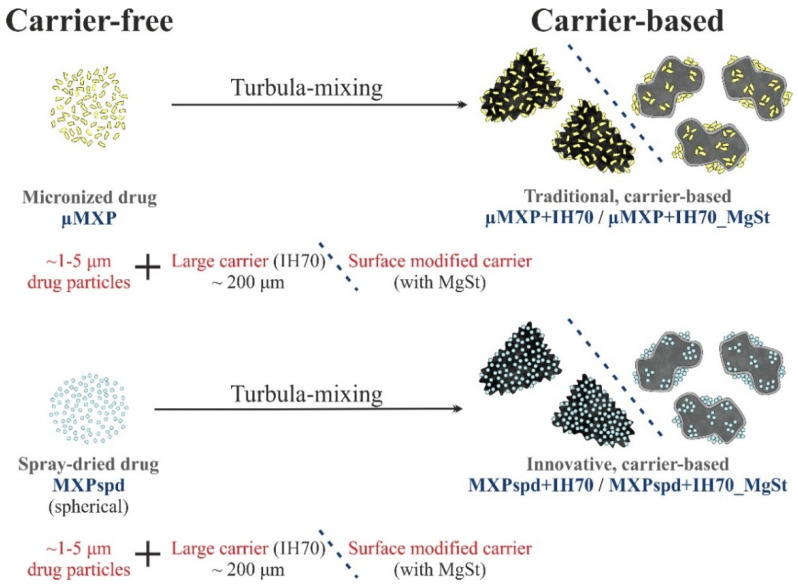
Schematic overview of the preparation.

Figure 1 presents the methods to produce the formulations of the carrier-free and carrier-based (traditional/innovative) DPI samples. The carrier-free samples (μMXP and MXPspd) were prepared in two ways. In the case of μMXP (Figure 1, upper left part), the particle size of 1.5 g of the raw MXP was reduced via the application of the Planetary Ball Mill (PM 100 MA, Retsch GmbH, Haan, Germany), using 400 rpm for 120 min; then, we manually sieved the sample through a 25 µm sieve (Retsch GmbH, Haan, Germany) for half an hour. The yield was 47.35 ± 0.87% after milling and sieving. In the other case (Figure 1, lower left part), spray drying was carried out to form the MXPspd, whereby 0.50 g of raw MXP was dissolved in a mixture of 89.5 g distilled water and 10.0 g 96% ethanol, heated to 80 °C [43].

During spray drying, a Büchi Mini Spray Dryer B-191 (Büchi Labortechnik AG, Flawil, Switzerland) was used, based on our previous research [28]. The following main setting parameters were applied: the set temperature of the drying air (T_in_) was 140 °C; the outlet temperature (T_out_) was 78 °C, which is below the melting point of MXP; the drying air flow rate (Asp.) was 75%; the sample pump speed (Pump) was 5%, and the compressed air flow rate (Airflow) was 600 L/h. The yield was 54.3 ± 1.4%. The carrier-based DPI formulations were prepared using the two abovementioned carrier-free MXP formulations (μMXP and MXPspd) and carrier/surface-modified carrier, blended in a 1:10 [51] mass ratio by Turbula mixing (T2F Turbula System Schatz; Willy A. Bachofen AG Maschinenfabrik, Basel, Switzerland) with a 30 min blending time at 60 rpm [52]. The powder mixtures were prepared according to the rules of powder mixing before Turbula mixing. The surface modification of the carrier (IH70) was carried out with the application of 2.0 w/w% of MgSt compared to the final formulations—Table 1 [25,53]—with Turbula blending for 4 h [22]. Thus, MgSt can form a thin film coating the surface of the IH, which can modify its surface properties [22].

#### 2.2.2. Blend Uniformity and Real Drug Content

After the production of the samples, homogeneity and drug content tests were carried out in case of the carrier-based formulations due to the use of mixing operations. The United States Pharmacopeia described that the investigations must be performed with DPI dosage units [54]. The inhalation dose of MXP is 1.3 mg, which corresponds to one-tenth of the MXP’s oral dose [28]. In each case, 14.3 ± 0.5 mg of the sample was selected from ten random places [55]. Those were dissolved in 10 mL methanol + pH 7.4 phosphate buffer (60 + 40 *v/v*%), and the MXP content was determined by the UV/VIS spectrophotometer (ATIUNICAM UV/VIS Spectrophotometer, Cambridge, UK) at a 364 nm wavelength. We determined beforehand the linearity of the MXP in this medium at the wavelength mentioned above. The linearity of the calibration curve was y = 0.0418x. The unit of the slope is mL/µg. The LOD value of the MXP was 0.109 µg/mL and the LOQ of the MXP was 0.330 µg/mL in the methanol + pH 7.4 phosphate buffer (60 + 40 *v/v*%) background. The used excipients do not have remarkable absorption at this wavelength.

#### 2.2.3. X-ray Powder Diffraction (XRPD)

The structural characterization of the samples was conducted using a BRUKER D8 Advance X-ray powder diffractometer (Bruker AXS GmbH, Karlsruhe, Germany). The radiation source was Cu K λ_I_ radiation (λ = 1.5406 Å). The studied solid-state forms were scanned at 40 kV and 40 mA, and the angular range was 3°–40° 2-Theta, at a step time of 0.1 s/step and step size was 0.01°. X-ray calibration was performed with a silicon disc. DIFFRACT plus EVA software was applied to evaluate the results. The diffractograms were corrected by Kα2, smoothed, and evaluated after background removal.

#### 2.2.4. Particle Size Distribution

Laser diffraction was used to establish the particle size distribution of the samples (Malvern Mastersizer Scirocco 2000, Malvern Instruments Ltd., Worcestershire, UK). Approximately 0.5 g of the microcomposite was loaded into a feeder tray. The dry analysis method was used, so the air was the dispersion media for the examined particles. The dispersion air pressure was set to 2.0 bars in order to determine whether particle attrition had occurred. Three parallel measurements were implemented. The particle size distribution was characterized by the D (0.1), D (0.5), and D (0.9) values.

#### 2.2.5. Scanning Electron Microscopy (SEM)

Investigation of the shape, surface characteristics, and approximate size of the samples was conducted by scanning electron microscopy (SEM) (Hitachi S4700, Hitachi Scientific Ltd., Tokyo, Japan). For the induction of electric conductivity on the surface of the samples, a sputter coater was used (Bio-Rad SC 502, VG Microtech, Uckfield, UK). The applied air pressure was 1.3–13.0 MPa. The coating of the samples happened with gold-palladium (90 s) under an argon atmosphere using a gold sputter module in a high vacuum evaporator.

#### 2.2.6. Interparticle Interactions

The contact angle (Θ) was determined by applying a Dataphysics OCA 20 apparatus (Dataphysics Inc. GmbH, Germany). The pastilles of the formulations were pressed from 0.10 g material with a 1 ton compression force (Perkin Elmer hydraulic press, Waltham, USA). All samples were measured in triplicate. It means that three pastilles per sample were dripped with polar liquid (distilled water) and the other three pastilles were dripped with dispersion liquid (diiodomethane). At the same time as the dropping, we made a recording by setting the apparatus to 1–25 s time interval; thereby, the detection and determination of the change of the contact angle were possible. Thus, we obtained the contact angle—always in the same second—of the two different applied fluids. The surface free energy $\left(\gamma_{s}\right)$ of the samples—which consists of two parts: a disperse part ($\gamma_{s}^{d})$ and a polar part ($\gamma_{s}^{p})$, so $\left(\gamma_{s}=\gamma_{s}^{d}+\gamma_{s}^{p}\right)$—was determined based on the Wu equation. The surface tension of the used liquids is known from the literature $\left(\gamma_{l}=\gamma_{l}^{d}+\gamma_{l}^{p}\right)$: distilled water $\gamma^{p}$= 50.2 mN/m, $\gamma^{d}$= 22.6 mN/m; and diiodomethane $\gamma^{p}$= 1.8 mN/m, $\gamma^{d}$= 49 mN/m [56]. There are only two unknowns in the Wu equation [57], the disperse ($\gamma_{s}^{d})$ and the polar component ($\gamma_{s}^{p})$ of the tested materials, which can already be expressed:

(1) $$\left(1+\cos\Theta \right)\gamma_{l}=\frac{4\left(\gamma_{s}^{d}\gamma_{l}^{d}\right)}{\gamma_{s}^{d}+\gamma_{l}^{d}}+\frac{4\left(\gamma_{s}^{p}\gamma_{l}^{p}\right)}{\gamma_{s}^{p}+\gamma_{l}^{p}}$$

where Θ = contact angle; γ = surface free energy; s = solid phase; l = liquid phase; d = dispersion component; and p = polar component.

The cohesion work (W_c_) is determined as the double of the surface free energy [58]:

(2) $$W_{c}=2\times \gamma_{s}$$

The adhesion work (W_adh_) can be formed between the two different materials (represented by numbers 1 and 2), and it can be obtained from the dispersion ($\gamma_{s}^{d})$ and the polar component ($\gamma_{s}^{p})$ values of the material, in this formula as $\gamma^{d}$ and $\gamma^{p}$. The adhesion work equals [58]:

(3) $$W_{\mathrm{adh}}=4\left[\frac{\gamma_{1}^{d}\gamma_{2}^{d}}{\gamma_{1}^{d}+\gamma_{2}^{d}}+\frac{\gamma_{1}^{p}\gamma_{2}^{p}}{\gamma_{1}^{p}+\gamma_{2}^{p}}\right]$$

The adhesion force (F_adh_) was calculated by Derjaguin’s approach [57]:

(4) $$F_{\mathrm{adh}}=2\pi \left(\frac{R_{A}R_{B}}{R_{A}+R_{B}}\right)W_{\mathrm{adh}}$$

where R_A_ and R_B_ are the radii of the A and B particles; those between the adhesive interactions were investigated. R was defined as D (0.5)/2, which was obtained during the particle size analysis.

The spreading coefficient (S_12_)—which is a dimensionless value—shows the probability of one material—1—on the surface of the other material—2. It is applied in binary systems to characterize the distribution. The spreading is favorable if the value is positive, and the number is high. In this study, the spreading of the MXP particles can be characterized on the surface of the carrier or the surface-modified carrier. The spreading coefficient or the reverse case can be obtained by applying the following equations [57,58]:

(5) $$S_{12}=4\left[\frac{\gamma_{1}^{d}\gamma_{2}^{d}}{\gamma_{1}^{d}+\gamma_{2}^{d}}+\frac{\gamma_{1}^{p}\gamma_{2}^{p}}{\gamma_{1}^{p}+\gamma_{2}^{p}}-\frac{\gamma_{1}}{2}\right]$$

(6) $$S_{21}=4\left[\frac{\gamma_{1}^{d}\gamma_{2}^{d}}{\gamma_{1}^{d}+\gamma_{2}^{d}}+\frac{\gamma_{1}^{p}\gamma_{2}^{p}}{\gamma_{1}^{p}+\gamma_{2}^{p}}-\frac{\gamma_{2}}{2}\right]$$

where $\gamma^{d}$ is the dispersed part of surface free energy, $\gamma^{p}$ is the polar part of surface free energy, and γ is the total surface free energy of those components that are spread on the other component [58]. We always applied Equation (6) in our work.

#### 2.2.7. In Vitro Aerodynamic Investigation

The Andersen Cascade Impactor (ACI) (Copley Scientific Ltd., Nottingham, UK) was applied to determine the aerodynamic particle size distribution (APSD) of the samples. This apparatus is authorized by the European Pharmacopoeia 2.9.18 /Method Chapter/ United States Pharmacopeia /Test Chapter <601>/ and by the Chinese Pharmacopoeia /Chapter <0951>/ to study the APSD [59]. During the measurements, a 28.3 ± 1 L/min flow rate was created by a vacuum pump (High-capacity Pump Model HCP5, Critical Flow Controller Model TPK, Copley Scientific Ltd., Nottingham, UK), and was measured by a mass flow meter (Flow Meter Model DFM 2000, Copley Scientific Ltd., Nottingham, UK). Before the in vitro inhalation tests, the eight collection plates of the ACI were coated with a Span^®^ 80 and cyclohexane (1 + 99 *w/w*%) mixture, so it was possible to repeat the inhalation into the ACI. The amounts filled into the capsules were calculated, the MXP content per sample was 1.3 mg [43]. By the investigation, two cycles of inhalation were applied for 4 s. Breezhaler^®^ (Novartis International AG, Basel, Switzerland) was used as a DPI device with three [60] transparent, size 3 hard gelatin capsules (Coni-Snap^®^, Capsugel, Bornem, Belgium). The applied DPI device, the capsules, the mouthpiece, the induction port, the eight plates of the ACI, and the filter were washed with methanol + pH 7.4 phosphate buffer (60 + 40 *v/v*%). The MXP concentration was determined by an ultraviolet-visible spectrophotometer (ATI-UNICAM UV/VIS Spectrophotometer, Cambridge, UK) at 364 nm wavelengths. By knowing the amount of the MXP in the washed elements, the emitted fraction (EF), the fine particle fraction (FPF), and the mass median aerodynamic diameter (MMAD) were calculated. The EF was defined as the percentage of the active ingredient found in the cascade impactor items (except the MXP found in the DPI device and capsules). The FPF represents the number of drug particles with aerodynamic diameter below 5 microns (FPF < 5 µm). Nevertheless, it is advisable to express the percentage of the particles less than 3 microns (FPF < 3 µm) as those can be deposited mainly in the deep lung. MMAD is expressed as that diameter of the particles deposited in the impactor for which 50 *w/w*% of the particles have a lower and 50 *w/w*% have a higher diameter [61]. The FPF and MMAD values were determined with the help of the KaleidaGraph 4.0 (Synergy Software, Reading, PA, USA), and a plot of the cumulative percentage undersize of the drug on a log probability scale against the effective cut-off diameter (ECD) was formed [62]. The calculation of the aerodynamic values is detailed in Appendix A.

#### 2.2.8. In Silico Assessment

The quantification of airway deposition of the inhaled drug was performed by using the Stochastic Lung Model (SLM). SLM is a whole respiratory tract particle deposition model initially developed by Koblinger and Hofmann (1990) [63]. This particle transport and deposition simulation tool have been under continuous development for almost three decades. In this work, the most up-to-date version of the model was applied, which was validated for the case of medical aerosols. In the upper airways (mouth cavity, pharynx, and larynx) the efficiency of the particle deposition is assessed by the help of the empirical deposition formulas derived from Cheng (2003) [64] and integrated into the SLM model. The geometry of the bronchial and bronchiolar parts is built up using randomly selected morphometrical parameters of the branching tubes (lengths, diameters, branching, and gravity angles) from distributions based on measurements on the airway casts (Raabe et al. 1976) [65]. The digital replica of the acinar airways is based on the anatomical description made by Haefeli-Bleuer and Weibel (1988) [66]. In this stochastic intrathoracic airway structure, the particles are tracked until they will be deposited or exhaled. In this part of the respiratory tract, the particle deposition is computed based on analytical deposition formulas deduced for straight and bent tubes and hemispheres. The main deposition mechanisms are the inertial impaction and the gravitational settling. Due to the size distribution of the aerosol drugs, Brownian diffusion is not significantly influencing their deposition. The main inputs of the deposition model were the breathing parameters of the patients and the APSDs of the considered drugs. The parameters characterizing the breathing of patients during the inhalation of the drug were adopted from the work of Colthorpe et al. (2013) [67]. They registered the breathing profiles of patients inhaling through the Breezhaler^®^ DPI. The aerodynamic size distributions of the particles were measured by impactor techniques.

#### 2.2.9. Release Assay

Dissolution studies were performed in the case of raw MXP, µMXP, and MXPspd. The last two formulations can be found on the carrier surface of the carrier-based samples. The tests were performed in simulated lung fluid—SLF (pH 7.4)—containing the following components in 900 mL: NaCl 0.68 g L^−1^, NaHCO_3_ 2.27 g L^−1^, Gly 0.37 g L^−1^, NaH_2_PO_4_ H_2_O 0.16 g L^−1^, CaCl_2_ 0.02 g L^−1^, and H_2_SO_4_ 5 mL 0.1 M [61]. Dissolution assays were performed under controlled conditions in the beakers using 30 mg of the sample in 2.5 mL of SLF, at 50 rpm [68] stirring, at 7 measurement times, and applying a 0.45 µm [61] pore size syringe filter (Nantong FilterBio Membrane Co., Ltd., Nantong, China). All samples were measured in triplicate. The amount of dissolved MXP was determined by the UV/VIS spectrophotometer (ATIUNICAM UV/VIS Spectrophotometer, Cambridge, UK) at 362 nm wavelengths. The linearity of the calibration curve of the MXP in the SLF was y = 0.0426x. The unit of the slope is mL/µg. The LOD of the MXP was 0.093 µg/mL, and the LOQ of the MXP was 0.281 µg/mL in the SLF background.

#### 2.2.10. Statistical Analyses

Statistical analyses were implemented using t-test calculations at the 0.05 significance level and with a one-tailed hypothesis, using Social Science Statistics available online [69]. All reported data imply ± SD of three parallel investigations (*n* = 3).

## 3. Results and Discussion

### 3.1. Blend Uniformity and Drug Content

The mixing uniformity of the DPIs should meet the criteria of the United States Pharmacopeia, which order the content uniformity to be between 85 and 115%, and the relative standard deviation of 10 dosage units to be ≤6%. The industrial standard is generally more stringent, often set between 90 and 110% [54]. Our carrier-based formulations also meet the latter more stringent criteria in the blending uniformity test; thus, homogeneity can be assumed (SD < 5%) [70]. Based on the calculated drug content, the exact amount of the compositions was determined to be filled into the capsules for performing the in vitro study.

### 3.2. Structural Investigations

**Figure 2:**
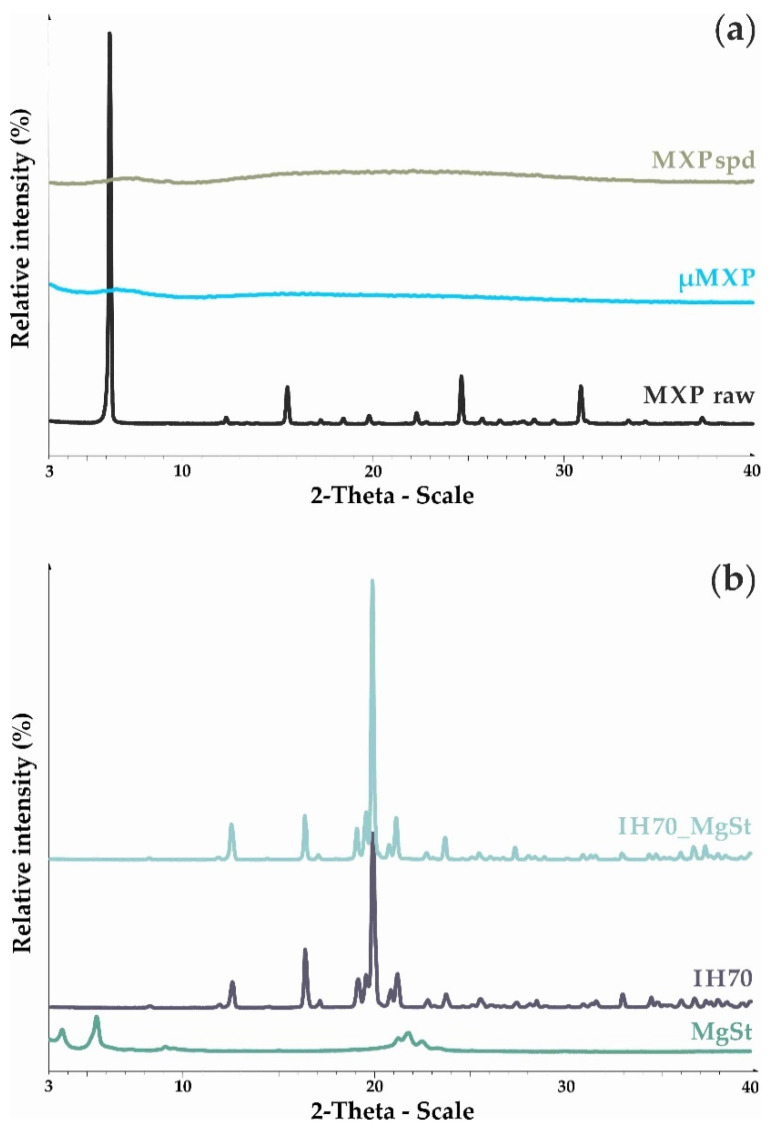
XRPD patterns of raw MXP, the carrier-free drug formulations (**a**), and the excipients (**b**).

Investigation of the structural character of the applied drug and used excipients are also crucial for the understanding of their aerodynamic behavior, efficacy, compatibility of the API and the excipient, and the stability of the dosage form. XRPD studies provided useful information on the structure and crystallinity state of the mechanically micronized (μMXP) and the spray-dried (MXPspd) drug, which was applied in the studied samples. Based on the XRPD patterns (Figure 2a), the crystalline nature of the raw MXP was confirmed. MXP peaks were detected at the following 2Θ values: 6.04, 15.35, 24.52, and 30.94. In the case of µMXP and MXPspd, no peaks were observed according to the XRPD patterns, which reflected the amorphous nature of these formulations. After the micronization process, the crystallinity of the API broke down, and after the spray-drying, its crystalline structure did not build up again (because of the fast evaporation of the solvent); so, an amorphous state was achieved. The characteristic peaks of the excipients (Figure 2b) were investigated, too. These peaks were determined for MgSt at 3.8 and 5.5 2-Theta degrees and for IH70 at 12.8, 16.8, and 20.0 2-Theta degrees. In the case of the surface-treated carrier (IH70_MgSt), no structural change was detected compared to IH70.

### 3.3. Particle Size Analysis and Scanning Electron Microscopy (SEM)

Particle size distribution and SEM images of the raw MXP, the carrier-free drug formulations, and the IH70 were carried out (Table 2). Based on the SEM images, the raw MXP sample contains big column-shaped crystals with a rough surface. This habit is not suitable for pulmonary application—D (0.5): 52.27 µm—because only API particles with 1–5 micron diameters are optimal to form depositions in the tracheobronchial region. Bigger particles than the above-indicated size range are deposited predominantly in the upper respiratory tract, while particles below 1 micron are mainly exhaled [71]. The μMXP consists of particles with an uneven surface and different morphology. The average particle size—D (0.5): 3.602 µm—is already recommendable for pulmonary drug delivery; however, the particle size distribution shows a relatively large range and the D (0.9) value already predicts deposition in the upper respiratory tract.

**Figure 3:**
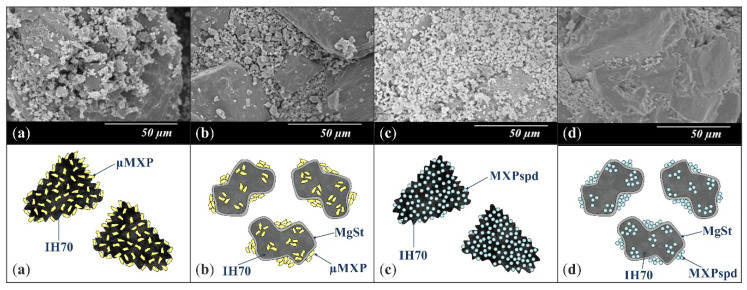
SEM recordings and schematic pictures of the carrier-based samples: (**a**) µMXP + IH70, (**b**) µMXP + IH70_MgSt, (**c**) MXPspd + IH70, and (**d**) MXPspd + IH70_MgSt.

MXPspd is a monodispersed sample with spherical morphology and contains dimples on the surface of the particles; furthermore, its particle size distribution is more favorable than that of the μMXP. The spherical morphology is beneficial, resulting in a smaller surface contact of particles of the formulation [72], which may also affect cohesion work. In the case of IH 70, which is a big carrier, a relatively smooth surface and columnar, near rhomboidal particles—D (0.5): 215.00 µm—were seen. SEM images (Figure 3) were also carried out to observe how MgSt modifies the surface properties of the IH70, and thus its role in the formulation. Remarkable differences were determined when the μMXP and MXPspd formulations were placed on the surface of IH70 and IH70_MgSt, while the uniform distribution of the drug particles—on the surface of IH70—was observed without surface modification of the carrier (Figure 3a,c). However, when MgSt was used for the surface treatment, the API particles did not uniformly coat the carrier and were present at higher concentrations in certain locations (Figure 3b,d). This phenomenon can also modify the interparticle interactions and thus affect the aerodynamic results.

### 3.4. Interparticle Interactions

The contact angle (Θ) (Table 3) of the raw and the applied materials were also detected using distilled water and diiodomethane, and the surface free energy $\left(\gamma_{s}\right)$ was obtained. Furthermore, the polarity and the cohesion work (W_c_) was calculated from these data. Table 3 shows that the highest polarity values were obtained in the case of the MXPspd. The polarity of the IH70_MgSt was lower than that of the IH70; in turn, the cohesion work (W_c_) of the MXPspd is smaller than that of the µMXP.

The adhesion work (W_adh_), the adhesion force (F_adh_) of the carrier-based formulations, and their characteristic spreading coefficient (S_21_) were determined (Table 4). The F_adh_ between the drug particles and the carrier/surface treated carrier is remarkably less in the case of MXPspd-containing carrier-based samples (MXPspd + IH70, MXPspd + IH70_MgSt), approximately half than for the μMXP-containing formulations (µMXP + IH70, µMXP + IH70_MgSt), which may result in more favorable aerodynamic values in the case of the former samples. It is assumed that the spherical morphology of MXPspd contributes to this result because these particles can contact the IH70/IH70_MgSt on smaller surfaces than the µMXP particles, and the F_adh_ calculation using Equation (4) described in Section 2.2.6 shows that the larger API particle size contributes to the greater F_adh_. This statement is also justified the high F_adh_ in the case of µMXP. It can also be stated that the presence of MgSt decreased the W_adh_ and F_adh_ of µMXP + IH70_MgSt and MXPspd + IH70_MgSt, which may also contribute to the improvement of the aerodynamic results. In addition, due to the negative numerical value, the spread of the API particles on the surface of the surface-modified carrier is less favorable in the case of µMXP + IH70_MgSt and MXPspd + IH70_MgSt, which has already been confirmed by the SEM recordings as well.

### 3.5. In Vitro Aerodynamic Assessment

From the FPF < 5 µm, the FPF < 3 µm, the MMAD, and EF values that are shown in Table 5, which were obtained from the in vitro lung model measurements described in Section 2.2.7, it can be stated that the conclusions made from the previous measurement results (structure, particle size distribution, morphology, and interparticle interactions) were appropriate. In case of the samples containing MXPspd, the drug particles have an amorphous structure, spherical morphology, optimal particle size and particle size distribution, as well as a low F_adh_ value, especially for the MXPspd + IH70_MgSt mixture—whereby the API particles can easily drift down during the inhalation from the surface of the carrier—resulting in remarkably better lung deposition results than for the µMXP-containing formulations. Although the µMXP has a wider particle size distribution than the 1–5 micron range, its morphology indicates that the drug particles can make contact on a larger surface than the MXPspd particles, and this has been shown slightly by the W_c_ values as well. However, the MMAD data show much higher values than the average particle size of µMXP—to which the 28.3 L/min test airflow may have contributed—and indicates that the particles can aggregate easier. These MMADs of the µMXP-containing formulations are already unfavorable because they are above 5 microns. These all contributed to the low FPF values, and the application of the carrier (µMXP + IH70) could not improve these results because the F_adh_ between the drug and the carrier particles is also high. The surface treatment of the carrier with MgSt could slightly improve the lung deposition results (µMXP + IH70_MgSt). In addition, the pulmonary deposition results of the µMXP + IH70 and µMXP + IH70_MgSt formulations, as traditional carrier-based DPI systems, were correlated with the FPF (<5 µm) results (20–30%) of the most of the marketed formulations mentioned in the introduction. Thus, the lung deposition results of the formulations containing MXPspd indicate that the spray-dried drug applied on the MgSt-treated carrier particles shows a remarkable improvement in FPF < 5 µm (72.32%) and FPF < 3 µm (46.05%) values over the carrier-free MXPspd formulation and especially over the µMXP and the traditional carrier-based samples. Furthermore, the presence of MgSt improved the interparticle interactions in the case of the MXPspd + IH70_MgSt sample compared to the MXPspd + IH70 formulation, which explains the notable difference in the lung deposition result. In terms of EF, the innovative, carrier-based formulations correspond to the requirement that the value must be between 85 and 115%, as measured by the APSD testing [71].

### 3.6. In Silico Test

**Figure 4:**
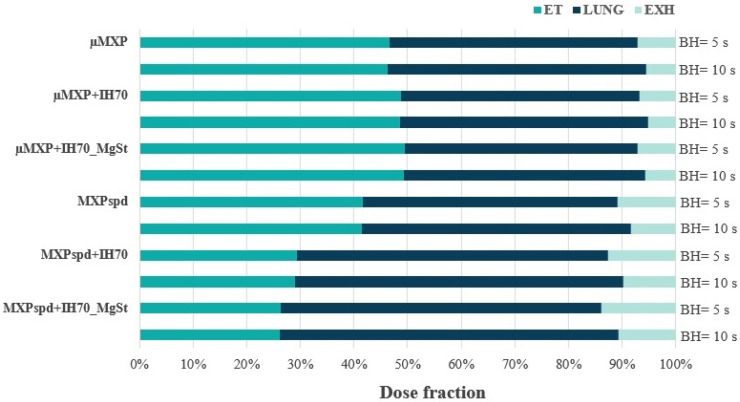
In silico simulation results of the studied DPI formulations (ET: extrathoracic airways; LUNG: bronchial and acinar parts; EXH: exhalation fraction).

The in vitro ACI lung model is applicable for comparing the DPI formulations; however, this is a non-disease-specific model and has defined ECDs for a given L/min, while providing no information on the exhaled (EXH) fraction. However, it connects well to the in silico investigations; so, we used the obtained data from the in vitro measurements according to the SLM method described in Section 2.2.8., where we applied the COPD-specific parameters (inhaled air volume: 1.7 L, inhalation time: 3.2 s, breath-hold time (BH) after the inhalation: 5 s and 10 s, exhalation time: 3 s) based on Colthorpe et al. [67]. Based on the in silico results (Figure 4), the obtained values correlated with the trends, which were determined by the in vitro measurements. So, the mechanically micronized drug (µMXP) blended on the carrier or the surface-treated carrier did not achieve a high level of lung deposition and therefore resulted in a remarkable extrathoracic (ET) deposition. In the case of MXPspd, mixing with IH or IH_MgSt shows further improvement in ET and LUNG values. Nevertheless, the EXH results were slightly higher in the case of the MXPspd-containing formulations than in the samples containing µMXP, which could be traced back to the differences in the average particle size, the particle size distribution, and the MMAD values between the MXPspd and the µMXP particles. While all of these results were small/narrow ranges of samples containing MXPspd, they showed a slightly higher EXH. However, with the 10 s BH the EXH values decreased, while the LUNG values improved with the 5 s BH in all samples. After all, the EXH values were reduced when the 10 s BH was used, thereby improving the LUNG values compared to the usage of the 5 s BH in each sample.

### 3.7. Release Assay Test Results

**Figure 5:**
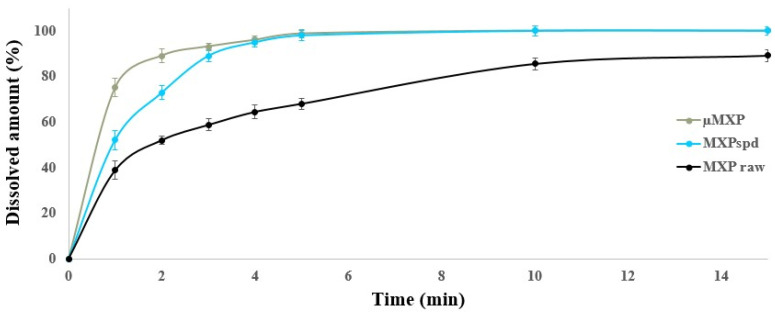
Dissolution test results of the raw MXP, µMXP, and MXPspd.

The results of dissolution studies of the raw MXP, µMXP, and MXPspd made under controlled conditions in SLF are illustrated in Figure 5. Based on the results, it can be stated that µMXP and MXPspd were almost 100% dissolved after 5 min. However, for raw MXP, an 80% release was achieved until the end of the test. The dissolution behavior of the samples can be closely related to the average particle sizes (D (0.5)) and the particle size distributions. After all, the raw MXP has a large D (0.5) value and also has a wide particle size distribution, and the D (0.5) of the µMXP is already close to that of the MXPspd, but the particle size distribution is much broader in the former case. Furthermore, the morphology and the W_c_ of the particles, as well as their aerosolization behavior, also predict easier agglomeration, which may contribute to the fact that the dissolution is less instantaneous in this sample. The physical properties of the MXPspd (D (0.5), particle size distribution, morphology, and W_c_) result in the best dissolution results among the compared samples and this sample has the best in vitro lung deposition and in silico results.

## 4. Conclusions

It can be concluded that in the case of a traditional, carrier-based system, after the surface treatment with MgSt, the carrier particles did not show much improvement in the in vitro dissolution results, and the interactive physical blend of the spray-dried drug—MXPspd—with the untreated carrier particles did not represent a definite improvement over the results of the MXPspd considering the in vitro and in silico data. The study shows that among the developed and innovative, carrier-based systems, the MXPspd + IH70_MgSt formulation had the most outstanding in vitro lung deposition results—FPF < 5 µm (72.32%) and FPF < 3 µm (46.05%)—which was also supported by in silico measurements and predicted by physical examinations. It means that the interparticle interactions and the micrometric properties of the drug particles have a major role in the final efficiency of the formulations, and they also affect the dissolution results.

Overall, in accordance with our goals, we managed to develop an innovative, carrier-based product with outstanding lung deposition values in the case of MXP, which meets the requirements of the DPIs and even achieved a ~70% FPF (<5 µm) result, similar to our previous developments [28,29]. It can be stated that in addition to the development of the carrier-free DPI systems, which currently have more focus at the international level—for example, our research team has successful carrier-free solutions for MXP and MX [43,44]—there are still opportunities for the enhancement of the carrier-based DPIs. Our present formulation, which is also useful for MXP and CIP, may even help to improve the effectiveness of CF and COPD therapy in the future. Moreover, we plan to produce a combined product containing these drugs, using our abovementioned formulation experience, because MXP can directly inhibit inflammation and CIP can directly inhibit bacterial infection; in addition, both can indirectly decrease the progression of the diseases by interfering with the pathophysiological cycle.

## Figures and Tables

**Table 1:** The composition of the prepared dry powder inhalation (DPI) samples.

Samples	µMXP	MXPspd	IH70	MgSt
µMXP	X	-	-	-
µMXP + IH70	0.2 g	-	2.0 g	-
µMXP + IH70_MgSt	0.2 g	-	1.956 g	0.044 g
MXPspd	-	X	-	-
MXPspd + IH70	-	0.2 g	2.0 g	-
MXPspd + IH70_MgSt	-	0.2 g	1.956 g	0.044 g

**Table 2:** Particle size distribution and morphology of the raw MXP, µMXP, MXPspd, and IH 70.

Samples	MXP Raw			µMXP			MXPspd			IH70		
**SEM** **pictures**	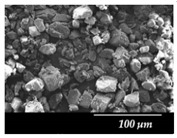			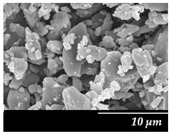			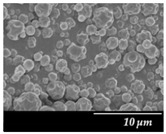			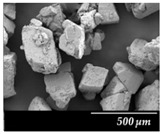		
**Particle size distribution**	D (0.1) (µm)	D (0.5) (µm)	D (0.9) (µm)	D (0.1) (µm)	D (0.5) (µm)	D (0.9) (µm)	D (0.1) (µm)	D (0.5) (µm)	D (0.9) (µm)	D (0.1) (µm)	D (0.5) (µm)	D (0.9) (µm)
	3.149	52.268	933.754	1.377	3.602	8.660	1.121	2.109	3.932	135.02	215.00	305.34

**Table 3:** Contact angles in the case of distilled water and diiodomethane, surface free energy and their components, polarity, and cohesion work of the applied material in the formulations.

Materials	Θ_water_ (°)	Θ_diiodomethane_ (°)	$\gamma_{s}^{d}$ (mN/m)	$\gamma_{s}^{p}$ (mN/m)	$\gamma_{s}$ (mN/m)	Polarity (%)	W_c_ (mN/m)
µMXP	25.13	23.53	42.07	33.18	75.25	44.09	150.50
MXPspd	26.40	29.90	39.93	33.44	73.37	45.58	146.74
IH70	3.30	6.00	45.58	36.88	82.46	44.72	164.92
IH70_MgSt	64.60	62.00	26.07	19.22	45.29	42.44	–
MgSt	102.63	68.64	24.33	2.64	26.96	9.79	53.92

**Table 4:** The work of adhesion, adhesion, and spread coefficient in the case of the carrier-based samples.

Products	W_adh_ (mN/m)	F_adh_ (mN)	S_21_
µMXP + IH70	104.98	1.168 × 10^−3^	6.87
µMXP + IH70_MgSt	76.55	0.849 × 10^−3^	−37.44
MXPspd + IH70	102.67	0.674 × 10^−3^	8.55
MXPspd + IH70_MgSt	76.80	0.493 × 10^−3^	−34.83

**Table 5:** Aerodynamic properties of the formulations.

Samples	FPF (%) < 5 μm	FPF (%) < 3 μm	MMAD (μm)	EF (%)
µMXP	27.71 ± 1.32	15.52 ± 0.66	6.54 ± 0.15	90.65 ± 1.43
µMXP + IH70	24.99 ± 0.89	14.71 ± 0.27	7.18 ± 0.06	92.30 ± 0.76
µMXP + IH70_MgSt	31.50 ± 1.08	19.17 ± 0.45	7.43 ± 0.11	72.06 ± 0.99
MXPspd	59.47 ± 1.33	37.66 ± 0.36	3.41 ± 0.18	70.74 ± 1.14
MXPspd + IH70	59.60 ± 0.65	35.68 ± 0.21	3.82 ± 0.16	86.40 ± 0.21
MXPspd + IH70_MgSt	72.32 ± 0.74	46.05 ± 0.41	3.11 ± 0.09	86.93 ± 0.78

**Figure A1:**
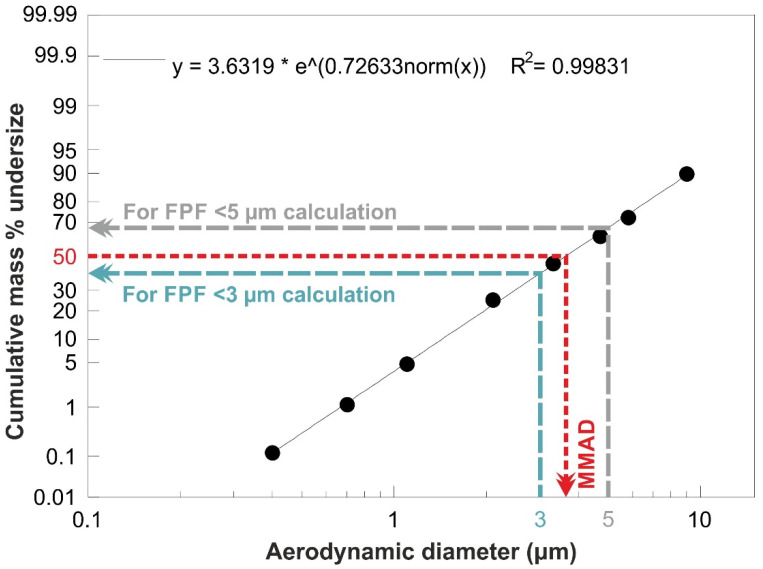
Curve from the KaleidaGraph 4.0 software, which was applied to the calculation of the aerodynamic properties.

## Appendix A

Supplement—Calculation of the aerodynamic values

After the in vitro aerosolization test (with ACI), the used DPI device, the capsules, the mouthpiece, the induction port (throat), the eight plates of the ACI, and the filter was washed with methanol + pH 7.4 phosphate buffer (60 + 40 *v/v*%). Applying the ultraviolet-visible spectrophotometer (ATI-UNICAM UV/VIS Spectrophotometer, Cambridge, UK) at 364 nm wavelengths, the amount of the MXP in the washed elements was calculated in the volume of the flasks, and by using the information on the dilution, absorbance, and slope of the calibration curve of the drug in the given medium. The emitted dose (ED) is the amount of deposited API in the ACI items (from the mouthpiece to the filter). It should be noted that if we want to express ED for a dose of MXP, we need to correct it by the number of the applied DPI capsules in the in vitro aerosolization test. However, we prefer the expression of EF, which was calculated as the ED in the percentage of the total collected drug; it is used in several publications [73,74]. We used the KaleidaGraph 4.0 (Synergy Software, Reading, PA, USA) to plot a log probability scale, where the ECDs (ACI, 28.3 L/min flow rate [59]) are given as the abscissa and the cumulative percentage less than the size range as the ordinate (Figure A1) [75].

The ordinate represents the cumulative percentage of the deposited drug mass collected from the stage (plate) 0 to the filter [75,76] of 5 µm aerodynamic diameter (Figure A1, grey line). The mass of the particles with a diameter of less than 5 µm can be calculated (this mass otherwise corresponds to the fine particle dose if divided by the number of the applied DPI capsules). A FPF < 5 µm can be obtained from the graph as a percentage of the ED [60]. From the percentage value for 3 µm (Figure A1, blue line) we can get the mass of the particles with a diameter less than 3 µm, from which we get FPF < 3 µm as a percentage of the ED. This value has also been expressed by several publications recently [77,78], because in the sub-tracheal region of the lung—in the deep lung—mainly particles below 3 µm form deposits [79]; thus, when comparing the aerodynamic properties of the DPI formulations, the calculation of the deep lung deposit value is also expedient. It should be noted that in addition to the reported results, the FPF < 4.7 µm calculation is always performed as a control, which is exemplified in the literature [80]. When we calculated the amount of drug deposited from stage 3 (in the case of ACI at 28.3 L/min flow rate, the size range of this stage is 3.3 µm–4.7 µm [81]) to the ED, the FPF < 4.7 µm was obtained as a percentage. While the results calculated from KaleidaGraph 4.0 (Synergy Software, Reading, PA, USA) in all cases of our measurements—an FPF < 5 µm—were realistically slightly higher, the published results were still supported. The MMAD is the abscissa value corresponding to the 50% value of the ordinate (Figure A1, red line), from which we get that diameter where 50 *w/w*% of the particles are larger and 50 *w/w*% are smaller [61,76].
